# Using regulatory enforcement theory to explain compliance with quality and patient safety regulations: the case of internal audits

**DOI:** 10.1186/s12913-018-2865-8

**Published:** 2018-01-30

**Authors:** Ulrike Weske, Paul Boselie, Elizabeth L. J. van Rensen, Margriet M. E. Schneider

**Affiliations:** 1 School of Governance, Utrecht University, Bijlhouwerstraat 6, 3511 ZC Utrecht, The Netherlands; 2 Quality & Patient Safety Department, University Medical Center Utrecht, Heidelberglaan 100, 3584 CX Utrecht, The Netherlands; 3 Staff Department Executive Board, University Medical Center Utrecht, Heidelberglaan 100, 3584 CX Utrecht, The Netherlands; 4 President of the Executive Board, University Medical Center Utrecht, Heidelberglaan 100, 3584 CX Utrecht, The Netherlands

**Keywords:** Implementation, Compliance, Quality and patient safety, Hospitals, Enforcement style, Internal audit

## Abstract

**Background:**

Implementing an accredited quality and patient safety management system is inevitable for hospitals. Even in the case of an obligatory rule system, different approaches to implement such a system can be used: coercive (based on monitoring and threats of punishment) and catalytic (based on dialogue and suggestion). This study takes these different approaches as a starting point to explore whether and how implementation actions are linked to compliance. By doing so, this study aims to contribute to the knowledge on how to increase compliance with obligatory rules and regulations.

**Methods:**

The internal audit system (the ‘tracer system’) of a large Dutch academic hospital is used as a case to investigate different implementation approaches and their effect on compliance. This case allowed us to use a multi-actor and multi-method approach for data collection. Internal audits (*N* = 16) were observed, audit reports were analyzed, and semi-structured interviews were conducted with both the internal auditors (*N* = 23) and the ward leaders (*N* = 14) responsible for compliance. Framework analysis was used to analyze the data.

**Results:**

Although all auditors use catalytic enforcement actions, these do not lead to (intended) compliance of all ward leaders. Rather, the catalytic actions contribute to (intended) compliance of ward leaders that are motivated, whereas they do not for the ward leaders that are not motivated. For the motivated ward leaders, catalytic enforcement actions contribute to (intended) compliance by increasing ward leaders’ knowledge of the rules and how to comply with them.

**Conclusions:**

Our findings suggest that the effectiveness of implementation actions depends not only on the actions themselves, but also on the pre-existing motivation to comply. These findings imply that there is not one ‘best’ approach to the implementation of obligatory rules. Rather, the most effective approach depends on the willingness to comply with rules and regulations.

**Electronic supplementary material:**

The online version of this article (10.1186/s12913-018-2865-8) contains supplementary material, which is available to authorized users.

## Background

Growing concern about the quality of care has resulted in increasing interest in healthcare accreditation [1–3]. In recent years, many countries have passed legislation to regulate the extent and the nature of quality assurance activities. Often, this legislation includes compulsory implementation of an accredited Safety Management System. Accreditation is an evaluation process used to assess, promote and guarantee quality of care against predetermined quality and patient safety standards [4]. Compliance with these standards offers the public protection by ensuring that requirements of safe healthcare are met. For hospitals, compliance with quality and patient safety regulation is inevitable due to high external pressures.

Having to implement a rule-based system does not automatically determine the way in which the rules of such a system are implemented. Research by Mikkelsen and colleagues [5] shows that, even in the case of obligatory rules, different approaches to rule-implementation can be used: coercive (based on monitoring and threats of punishment) and catalytic (based on dialogue and suggestion). Their study indicates that a catalytic approach is related to higher levels of motivation, whereas a coercive approach is not. These findings suggest that hospitals can use different approaches to rule-implementation and that these approaches are related to distinct outcomes.

So far, research into the link between these implementation approaches and compliance in the organizational context has been limited. Therefore, this paper aims to explore whether and how coercive and catalytic approaches to implementation are related to compliance with rules and protocols. By doing so, this study contributes to the knowledge on effective implementation of obligatory rules in hospitals.

### Enforcement styles and compliance

To achieve compliance with quality and patient safety regulations that are part of internal compliance systems, a range of implementation – or enforcement – styles can be used. In broad terms, two very different ideal typical enforcement styles are at the basis of most enforcement research: catalytic and coercive [5–11]. A catalytic enforcement style is based on the assumption that individuals are motivated to comply with rules, but that they are not able to do so because they do not have the capacity to be compliant or they do not understand what can be done to be compliant. Compliance is encouraged through technical and financial support, education and other inducements. A coercive enforcement style is based on the assumption that individuals are unwilling to comply with regulations and that they must be compelled to do by imposing sanctions for those out of compliance. From this perspective, individuals comply because they fear the consequences of being found in violation. In the regulatory enforcement literature, it is argued that neither of the approaches should be overdone; an entirely persuasive approach runs the risk of amoral calculators who take advantage by breaking the rules, whereas an entirely coercive approach could lead to negative consequences such as decreasing involvement with regulation and the withholding of information [10].

## Methods

To investigate enforcement approaches and their effect on compliance, we used the internal audit and feedback system of a large Dutch academic hospital as a case. The internal audit and feedback system that is used within the hospital is based on the tracing approach of the Joint Commission International [12] (JCI). Whereas the main focus of the JCI audits is on controlling and judging, the tracer system of the hospital is focused on supporting the wards in their improvement. The tracers are executed by couples consisting of the hospitals’ employees (medical, nursing, and other), who all received training on the accreditation system, the specific hospital policies and on how to perform a tracer and how to give feedback to the audited wards. At the time of the data collection (February – May 2015), 62 internal audits were conducted by a group of 64 auditors.

A combination of qualitative research methods was used to investigate all actors involved in the audits. First, 16 randomly selected internal audits – performed by 30 different auditors – were observed. Based on the non-participatory observations, field notes were made that focused on the actions of the internal auditors and the reactions of the ward leaders. Second, the resulting audit reports were analyzed. Third, interviews were conducted with 23 internal auditors and 14 ward leaders. Five of the observed auditors were not interviewed since they had just started as an auditor and two were not willing to participate in an interview. 12 of the observed auditors had their primary job in patient care (3 doctors and 9 nurses), whereas 18 had supportive jobs (including policy advisors, and ICT and financial employees). We observed 19 female and 11 male auditors. In Additional file 1, an overview of the auditors per audit can be found. Of the 16 observed ward leaders, two did not respond to the invitation to participate in an interview.

The interviews were semi-structured and guided by a topic list based on our theoretical focus (see Additional file 2). Interviews with the internal auditors focused on reflecting on the enforcement actions used in the observed audit. Interviews with ward leaders focused on how they had perceived the audit and what actions they took or intended to take based on the audit results. Since the ward leaders are responsible for ensuring compliance of their subordinates, their (intended) actions are an important indicator of audit effectiveness. Interviews lasted between 30 minutes and one hour and were tape recorded and transcribed verbatim [13].

To analyze the data, the framework method was used [14, 15]. The framework method is a systematic method that allows for assessment of the data both across cases and themes. The first step was familiarization with the data by reading the whole observation reports, interview transcripts and audit reports. In addition, some interviews were coded open to ensure that important aspects of the data were not missed. After familiarization with the data, two steps of analysis that are central to the framework method were followed. In the first step, a framework of central themes and subthemes was created based on our central theoretical concepts. These themes included enforcement actions by internal auditors (with subthemes coercive and catalytic actions and reasons for these actions), ward leaders’ (intended) actions based on the internal audits (with subthemes actions and reasons for these actions). In the second step, a matrix was generated that summarizes these themes and subthemes for each internal audit. This matrix grouped together the data collected from all actors that were involved in a particular audit. This means that for each audit, the matrix displayed enforcement actions of the internal auditors (based on the observations, audit reports and interviews with the internal auditors) and ward leaders’ (intended) actions and reasons for these actions (based on the interviews with the ward leaders). During both steps, the findings were discussed with all authors and the final results were checked with a tracer expert of the hospital.

## Results

Most audits that were observed proceeded as follows. At the start of the audit, the internal auditors announced to the ward leader that the ward was going to be audited. Next, the auditors used observations, interviews with employees and patients, and information from patient files and documents to collect information on the ward’s compliance with standards and procedures. Based on this information, the auditors made an audit report in which they scored whether wards were compliant (green score) or non-compliant (orange or red score) on the separate quality and patient safety standards. After finishing the reports, the auditors provided feedback about the audit results to the ward leaders and published the audit report on the hospitals’ intranet. The results section focuses on the last step of the audit: the feedback provision to the ward leader.

### Enforcement style of internal auditors

The internal auditors focused on providing feedback to the wards on whether they were complying with the rules and regulations that are part of the internal audit. They did this by filling out an audit report about the ward and by communicating the scores of the audit report to the ward leaders. Overall, the auditors had a facilitative approach, aimed at supporting the wards in improving. Examples included showing understanding for wards that are non-compliant, providing tips and tricks (this included giving suggestions for implementation, showing where protocols can be found), clarifying policies, explaining the rationale behind or importance of policies, and asking ward leaders what their points of interest for the audit are. “Then I tell them, I know it is a difficult problem, also at my ward. And often I have suggestions about what I’ve seen at other wards or how we do it at my ward” (internal auditor #2). We did not observe any instances of (threats of) punishments by the internal auditors when performing the internal audit.

Small differences were found between auditors in how strict they were in their scoring. Some auditors gave a red score as soon as a ward was not fully compliant with a rule, whereas others gave more green or orange scores, even when wards were not (fully) compliant with rules. Despite these small differences in rule rigidity, auditors’ overall approach towards the ward leaders can be characterized as catalytic. A complete overview of auditors enforcement actions can be found in Additional file 3.

### Linking auditors’ enforcement approach and ward leaders’ (intended) actions

This leads to the question whether the overall catalytic enforcement actions of auditors lead to relatively uniform (intended) actions from ward leaders? About half of the ward leaders indicated to (have intentions to) take actions to improve compliance with standards and procedures. In some cases, ward leaders themselves took action, for example by discussing the results in a work meeting. In other cases, they used the ward’s ‘normal’ improvement structure to initiate changes. These improvement structures included actions such as assigning the improvement action to a senior nurse or using lean management strategies.

The other half of the ward leaders did not indicate to (have intentions to) take actions to improve compliance. Often, these ward leaders did not even look at the results of the audit. “I didn’t take a look at the results to be honest, so I don’t even know what the results were. I do remember it wasn’t really impressive” (ward leader #4). What stands out is that whereas all auditors used mainly catalytic actions, this led to (intended) actions of only half of the ward leaders. Since all auditors applied a catalytic approach, it needs to be noted that this study does not allow for an evaluation of the effect of a coercive audit approach.

### The role of ward leaders’ motivation to comply

So far, the findings indicate that the catalytic enforcement actions of auditors are linked to (intended) actions of only half of the ward leaders. This leads to the question why ward leaders react differently to similar enforcement actions of auditors.

One of the most important differences we found between compliant and non-compliant ward leaders was their motivation to comply with the standards and procedures of the accreditation. Ward leaders that indicated to (intent to) take actions had a more positive attitude towards the policies and regulations that are central to the internal audit. They argued that, overall, they understand the importance of the rules and how compliance with the rules contributes to higher levels of quality and patient safety. It is important to note that the ward leaders have been motivated before, and not as a result of, the internal audit. “And then JCI was introduced and, well, the rules made sense, so we agreed on implementing them. And from that moment on we try to keep the standard as high as possible, we set the bar high” (ward leader #5).

Ward leaders that did not (have the intention to) take actions, on the other hand, were not motivated to follow the rules and guidelines that are part of the accreditation. Often, these ward leaders indicated that they did not agree on the importance of the rule or protocol they should be compliant with. The argument was mostly that the rule was only there because the accreditation organization requires compliance, rather than as a means to improve the quality of care. As one ward leader argued: “I don’t really like the rules, I’m more about keeping it practical and workable and having high quality, but rules, well, rules are also there to deviate from them” (ward leader #4). In line with the ward leaders’ low motivation regarding the accreditation, the ward leaders did not consider the internal audits useful. According to these ward leaders, the issues that arose during the audit were not in line with the ward’s priorities for improvement.

### The role of audits in increasing capacity to comply

For the motivated ward leaders, the internal audits contributed to their capacity to comply by providing ward leaders with an overview of their level of compliance and concrete input and support for improvement: “It is helpful when people look at your ward critically and show you where you’re at (…). The results were very clear and it were concrete points that help us to improve” (ward leader #12). The audit results contributed to ward leaders’ capacity to comply since they often did not know that the ward was non-compliant or did not know how to correctly implement a rule or protocol. Since all auditors were catalytic in their approach, all audits contributed to ward leaders’ capacity to comply.

However, the auditors could not increase capacity in all situations. This was the case when there were structural factors that prevent improvements. Several wards could, for example, not comply with the rules for storage of (medical) equipment because they did not have sufficient storage room to comply with all standards and procedures. The internal auditors could not address these capacity issues.

## Discussion

The results indicate that all auditors use catalytic enforcement actions based on dialogue and suggestions. Whether these catalytic actions contribute to ward leaders’ (intended) improvement actions depends on the motivation of ward leaders: only motivated ward leaders indicate to (have intentions to) take improvement actions based on the audit results. For motivated ward leaders, the internal audits contribute to their capacity to comply by increasing their knowledge of the rules. These findings imply that the effectiveness of enforcement actions depends on the pre-existing motivation of ward leaders.

Our finding that a catalytic style does not lead to compliance in all cases is in line with previous enforcement studies [10, 16, 17]. These studies indicate that a purely catalytic approach might not increase compliance since it is necessary to ‘get tough’ up to a point. Without a certain degree of coercion, an entirely catalytic approach runs the risk of amoral calculators taking advantage of leniency by breaking the rules. Previous enforcement studies have provided support for the importance of coercion as a backstop for catalytic approaches [16, 18, 19].

The results of our study indicate that it depends on ward leaders’ motivation whether a catalytic approach is an effective means to increase compliance. This finding is also in line with enforcement studies, where it has been argued that the nature of the motivation determines which enforcement actions will be most effective [20]. If regulated entities are motivated to comply, catalytic actions are sufficient to make them comply; if regulated entities are not motivated to comply, coercion might be necessary to ensure compliance [20]. Based on responsive regulation theory [21, 22], catalytic actions should be used when cooperation is encountered and coercive actions should be used when persistent opposition is encountered [10].

Moreover, our findings indicate that catalytic enforcement actions contribute to compliance by increasing ward leaders’ capacity to comply. This finding is in line with previous enforcement studies [17] that have found an indirect link of catalytic enforcement actions with compliance via increased knowledge of rules. The role of capacity to comply suggests that willingness to comply is insufficient unless individuals are also aware what is desired to be compliant and are able to carry out what is necessary [23]. Based on the results of this study and the insights from enforcement theory, a catalytic approach is most effective when the audit focuses on increasing ward leaders’ knowledge of the rules and how to comply.

Our findings also indicate that catalytic actions do not always increase capacity to comply; this is the case when structural factors prevent improvements. Previous studies have found similar ‘barriers’ to improvement in cases where the financial capacity to comply is too low [23]. These findings imply that addressing issues that a ward cannot comply with due to capacity issues is not useful in the context of an internal audit. In this case, wider organizational interventions that are able to address these capacity issues are needed.

This study may have some limitations. First, a specific type of ‘internal audit’ was used as a vehicle for our investigation of enforcement actions and their effect on compliance. This specific vehicle might prohibit us from generalizing all of the factors that explain why enforcement leads to compliance. However, since most of our findings are in line with the enforcement literature, it is expected that our findings will still be valid in other contexts. Moreover, it allowed us to investigate specific enforcement actions with different methods (observations and interviews) and including all involved actors (auditors and ward leaders). Second, it was not possible to interview all involved ward leaders. This could have led to a selection bias, where especially the wards with positive attitudes towards the internal audits were selected. However, since some interviewed ward leaders were quite critical about the internal audits and the accreditation system of the hospital, no reason is seen to assume a selection bias. Moreover, the ward leaders were not informed about the specific goal of the research and the researcher collecting the data had an independent position. Third, the ‘outcome measure’ of (intended) actions of ward leaders could be unreliable, for example when ward leaders feel pressure to give socially responsible answers or when they over- or underestimate their own actions. However, we think this effect is limited since there were several ward leaders that indicated to take no actions at all, or only on certain items. Moreover, in the interviews, we asked for specific actions and examples, making socially desirable answers less likely.

## Conclusions

Catalytic enforcement actions based on dialogue and suggestions contribute to compliance with quality and patient safety rules and regulations by increasing capacity to comply. This is only the case, however, when ward leaders are motivated to comply with the rules and regulations. When ward leaders are not motivated, catalytic enforcement actions do not contribute to ward leaders’ compliance.

These findings are relevant for the knowledge on the successful implementation of obligatory rules and regulations since they suggest that the effectiveness of implementation actions depends not only on the actions themselves, but also on pre-existing motivation to comply. Our findings are important since they imply that there is not one ‘best’ approach to the implementation of rules in hospitals. Rather, the ‘best’ approach depends on the pre-existing willingness to comply with rules and regulations.

## Additional files

Additional file 1: Overview of Auditors. (DOCX 14 kb)

Additional file 2: Interview Guides. (DOCX 19 kb)

Additional file 3: Data sources for auditor enforcement style. (DOCX 18 kb)

## Abbreviations

JCI: Joint Commission International

## References

[1] Shaw CD, Kutryba B, Braithwaite J, Bedlicki M (2010). Sustainable healthcare accreditation : messages from Europe in 2009. Int J Qual Heal Care.

[2] Hinchcliff R, Greenfield D, Moldovan M, Westbrook JI, Pawsey M, Mumford V (2012). Narrative synthesis of health service accreditation literature. BMJ Qual Saf.

[3] Saltman RB, Busse R, Mossialos E (2002). Regulating entrepreneurial behavior in European health care systems.

[4] Al-Awa B, de Wever A, Melot A, Devreux I (2011). An overview of patient safety and accreditation : a literature review study. Res J Med Sci.

[5] Mikkelsen M, Jacobsen CB, Andersen LB (2017). Managing employee motivation: exploring the connections between managers’ enforcement actions, employee perceptions, and employee entrinsic motivation. Int Public Manag J.

[6] Reiss AJ, Hawkins K, Thomas J (1984). Selecting strategies of social control over organizational life. Enforcing regulation.

[7] Shover N, Lynxwiler J, Groce S, Clelland D, Hawikins K, Thomas J (1984). Regional variation in law enforcement: the surface mining control and reclamation act of 1977. Enforcing regulation.

[8] Hutter BM (1989). Variations in regulatory enforcement styles. Law Policy..

[9] Gormley WT (1998). Regulatory enforcement styles. Polit Res Q.

[10] Mascini P, Van Wijk E (2009). Responsive regulation at the Dutch food and consumer product safety authority : an empirical assessment of assumptions underlying the theory. Regul Gov.

[11] May PJ, Wood RS (2003). At the regulatory front lines: inspectors’ enforcement styles and regulatory compliance. J Public Adm Res Theory.

[12] Joint Commission International. www.jointcommission.org.

[13] Braun V, Clarke V. Using thematic analysis in psychology. Qual Res Psychol 2006;3 May 2015:77–101.

[14] Gale NK, Heath G, Cameron E, Rashid S, Redwood S (2013). Using the framework method for the analysis of qualitative data in multi-disciplinary health research. BMC Med Res Methodol.

[15] Moullin JC, Sabater-Hernández D, Benrimoj SI (2016). Qualitative study on the implementation of professional pharmacy services in Australian community pharmacies using framework analysis. BMC Health Serv Res.

[16] May PJ, Winter SC (1999). Regulatory enforcement and compliance: examining Danish agro-environmental policy. J Policy Anal Manag..

[17] May PJ, Winter SC, Parker C, Nielsen VL (2011). Regulatory enforcement styles and compliance. Explaining regulatory compliance: business responses to regulation.

[18] Braithwaite J. To punish or persuade: enforcement of coal mine safety: SUNY Press; 1985.

[19] Burby RJ, Paterson RG (1993). Improving compliance with state environmental regulations. J Policy Anal Manag..

[20] Kagan RA, Gunningham N, Thornton D, Parker C, Nielsen VL (2011). Fear, duty, and regulatory compliance: lessons from three research projects. Explaining compliance: business responses to regulation.

[21] Ayres I, Braithwaite J (1995). Responsive regulation: transcending the deregulation debate.

[22] Braithwaite V, Braithwaite J, Gibson D, Makkai T (1994). Regulatory styles motivational postures and nursing home compliance. Law Policy.

[23] Winter SC, May PJ (2001). Motivation for compliance with environmental regulations. J Policy Anal Manag.

