# Supplementary material for: Using regulatory enforcement theory to explain compliance with quality and patient safety regulations: the case of internal audits
Source: BMC Health Serv Res. 2018 Jan 30;18:62. doi: 10.1186/s12913-018-2865-8 (PMC5791382; doi:10.1186/s12913-018-2865-8)
Supplement: Supplementary file 1 — Overview of Auditors. (DOCX 14 kb) [file 12913_2018_2865_MOESM1_ESM.docx]

**Additional file I: overview of auditors**

| Internal audit | Auditors | | |
| --- | --- | --- | --- |
|  | Doctor | Nurse^1^ | Supportive^2^ |
| 1 + 15^3^ |  | 1 | 1 |
| 2 |  |  | 2 |
| 3 |  | 1 | 1 |
| 4 | 1 |  | 1 |
| 5 |  | 1 | 1 |
| 6 |  |  | 2 |
| 7 |  |  | 2 |
| 8 |  | 1 | 1 |
| 9 |  | 1^4^ | 1^4^ |
| 10 |  | 1^4^ | 1 |
| 11 |  | 1 | 1 |
| 12 | 1^4^ |  | 1^4^ |
| 13 | 1 |  | 1 |
| 14^4^ |  | 1^4^ | 1 |
| 16^4^ |  | 1 | 1^4^ |
| Total | 3 | 9 | 18 |

^1^ ‘Nurse’ includes nurses, nurse managers and paramedical professions.

^2^ ‘Supportive’ includes policy advisors, ICT advisors and financial employees.

^3^ Audits 1 and 15 were performed by the same couple of auditors

^4^ These auditors or ward leaders were not interviewed
