# Supplementary material for: Using regulatory enforcement theory to explain compliance with quality and patient safety regulations: the case of internal audits
Source: BMC Health Serv Res. 2018 Jan 30;18:62. doi: 10.1186/s12913-018-2865-8 (PMC5791382; doi:10.1186/s12913-018-2865-8)
Supplement: Supplementary file 2 — Interview Guides. (DOCX 19 kb) [file 12913_2018_2865_MOESM2_ESM.docx]

**Additional file II: topiclists**

**Interviews with internal auditors**

*Background*

- What is your function in the hospital?
- What is your vision on quality & safety within the hospital?
- What is your experience as an internal auditor?

*Audit style*

- What is your approach to audits in general?
- What was your approach in this specific audit?
  - Focused on (treats of) punishment?
  - Focused on providing support?

*Reasons for style*

- Is your approach always the same? Why (not)?
  - Differences between audited wards
  - Differences between rules

*Other*

- Are there any other factors that play a role in your execution of the audit?

**Interviews with ward leaders**

*Background*

- What is your function in the hospital?
- What is your vision on quality & safety within the hospital?
- What are your experiences with internal audits?

*Perception of audit*

- Can you tell something about your experience of the audit of [date]? Did anything stand out?
- How did you experience the approach of the auditors?
  - Focused on (treats of) punishment?
  - Focused on providing support?

*Behavior*

- Did you take actions or are you going to take actions based on the audit?
  - What actions?
  - How?
  - Why (not)?

*Other*

- Are there any other factors that play a role in your experience of the audit and whether you take actions based on the audit result?
