# Supplementary material for: Using regulatory enforcement theory to explain compliance with quality and patient safety regulations: the case of internal audits
Source: BMC Health Serv Res. 2018 Jan 30;18:62. doi: 10.1186/s12913-018-2865-8 (PMC5791382; doi:10.1186/s12913-018-2865-8)
Supplement: Supplementary file 3 — Data sources for auditor enforcement style. (DOCX 18 kb) [file 12913_2018_2865_MOESM3_ESM.docx]

**Additional file III: Data sources for auditor enforcement style**

| **Audit #** | **Strictness of scoring¹** | **Observation** | **Interviews** |
| --- | --- | --- | --- |
| 1 | 31% (39 scored: 7 red, 5 orange, 27 green) | Providing overview of compliance, scoring strict on important items, having a dialogue | Auditor 1) Providing overview of compliance, scoring strict on important items, clarifying items  Auditor 2) Providing tips and tricks, showing understanding, emphasizing importance of items |
| 2 | 11% (46 scored: 1 red, 4 orange, 41 green) | (no ward leader present) | Auditor 1) Having a dialogue, providing overview of compliance, not scoring too strict  Auditor 2) Providing overview of compliance, clarifying items |
| 3 | (no audit report available) | Providing overview of compliance, providing tips and tricks | Auditor 1) Not scoring too strict, clarifying items, providing overview of compliance  Auditor 2) Providing overview of compliance, providing tips and tricks |
| 4 | 4% (50 scored: 0 red, 2 orange, 48 green) | Emphasizing importance of items, clarifying items, showing understanding, not scoring too strict | Auditor 1 + 2) Providing overview of compliance |
| 5 | 8% (53 scored: 0 red, 4 orange, 49 green) | (no ward leader present) | Auditor 1) Providing overview of compliance, creating awareness  Auditor 2) Providing overview of compliance |
| 6 | 15% (47 scored: 4 red, 3 orange, 40 green) | Providing tips and tricks, scoring strict on important items, clarifying items, emphasizing importance of items | Auditor 1) Providing overview of compliance  Auditor 2) Clarifying items, scoring strict on important items, emphasizing importance of items |
| 7 | 9% (33 scored: 0 red, 3 orange, 30 green) | Asking ward leader for his/her points of interest, showing understanding | Auditor 1 + 2) Providing overview of compliance |
| 8 | 6% (16 scored: 0 red, 1 orange, 15 green) | (no ward leader present) | Auditor 1) Providing overview of compliance, showing understanding  Auditor 2) Providing overview of compliance |
| 9 | 35% (40 scored: 13 red, 1 orange, 26 green) | Asking ward leader for his/her points of interest, providing overview of compliance, emphasizing importance of items, clarifying items | (no interviews) |
| 10 | 22% (40 scored: 2 red, 7 orange, 31 green) | (no ward leader present) | Auditor 1) Providing overview of compliance  Auditor 2) (no interview) |
| 11 | 6% (54 scored: 2 red, 1 orange, 51 green) | Providing overview of compliance | Auditor 1 + 2) Providing overview of compliance |
| 12 | 20% (40 scored: 3 red, 5 orange, 32 green) | Asking ward leader for his/her points of interest , providing tips and tricks, providing overview of compliance, having a dialogue | (no interviews) |
| 13 | 48% (21 scored: 3 red, 7 orange, 11 green) | Showing understanding, not scoring too strict | Auditor 1) Creating awareness, scoring strict on important items  Auditor 2) Providing overview of compliance, not scoring too strict |
| 14 | 38% (47 scored: 16 red, 2 orange, 29 green) | (no ward leader present) | Auditor 1) Providing overview of compliance  Auditor 2) (no interview) |
| 15 | 24% (41 scored: 6 red, 4 orange, 31 green) | Providing overview of compliance, providing tips and tricks, scoring strict on important items | (same auditors as audit 1) |
| 16 | 10% (31 scored: 2 red, 1 orange, 28 green) | (no ward leader present) | Auditor 1) Providing overview of compliance, creating awareness  Auditor 2) (no interview) |

¹ This column shows the percentage of red or orange scores compared to the total number of items that was scored in the audit. The higher the percentage, the ‘stricter’ the audit.
